# Iatrogenic Hypermagnesemia in a Patient With Preeclampsia Caused by Misinterpretation of the Magnesium Reporting Unit Following Magnesium Sulfate Administration

**DOI:** 10.7759/cureus.32446

**Published:** 2022-12-12

**Authors:** Mohamed S Omer, Syed Latif, Ricky Grisson

**Affiliations:** 1 Pathology and Laboratory Medicine, Rhode Island Hospital, Brown University, Providence, USA

**Keywords:** clinical pathology, obstetrics and gynecology, clinical chemistry, preeclampsia, hypermagnesemia

## Abstract

Because of the lack of standardization, different laboratories report plasma magnesium sulfate or magnesium level in different units, leading to errors in diagnosis and case management; especially when a patient's workup was done in a hospital and then transferred to another hospital. Failure to recognize or understand the reporting units can confuse clinicians and healthcare workers, leading to erroneous interpretations and, consequently, to misdiagnosis of hypermagnesemia or hypomagnesemia.

In this report, we present a 32-year-old female patient at 31 weeks of gestation with a history of a multi-substance use disorder who was transferred to the hospital after taking 2.5mg of fentanyl. At the ER, her blood pressure and liver function tests were found to be high. Her albumin was 3.4 g/dl, aspartate transaminase (AST) was 104 IU/l, alanine transaminase (ALT) was 19 IU/l, and anaplastic lymphoma kinase (ALK) phosphatase was 148 IU/l; however, the renal function test was within the normal range. She had a few twitching movements. Her troponin I was high at 648 ng/L, and her B-type natriuretic peptide was 186.1 pg/mL, but her ECG showed a normal result. Magnesium was initiated on a 6 mg bolus, then 2 mg/dl due to preeclampsia concerns. She was transferred to another hospital ICU that used mEq/L as a reporting unit for magnesium and magnesium sulfate levels. The travel nurse did not alert the physician, thinking that magnesium sulfate was still within with therapeutic range of 4.8-8.4 mg/dL. However, considering 4.0-7.0 is the therapeutic range of magnesium sulfate utilizing mEq/L as a reporting unit instead of mg/dL, the patient was found to be at a supratherapeutic level, reaching up to 8.2 mEq/L. The physician discounted the magnesium sulfate. Her blood picture showed normocytic normochromic anemia. Her hemoglobin was 10.9 g/dl, her hematocrit was 33.8%, her mean corpuscular volume (MCV) was 92.5 fL, and her mean corpuscular hemoglobin (MCH) was 29.5 fL. Her platelets were within the normal range (169 10exp9/L). Her temperature was 99.6 F with increased white blood cells (17.7 10e9/L). The fetus was delivered via C-section on the third day of admission in the setting of persistently low fetal heart rates. The patient was extubated on the fourth day of admission and later transferred to the ward and discharged on the fifth day.

Monitoring the magnesium level while administrating magnesium sulfate is essential to avoid iatrogenic hypermagnesemia. Utilizing different units at the same laboratory or across different laboratories could lead to the healthcare providers misinterpreting the result, which can lead to misdiagnosing iatrogenic hypermagnesemia. Therefore, standardizing magnesium units across the Clinical Laboratory Improvement Amendments (CLIA) and College of American Pathologists (CAP)-accredited laboratories is highly recommended. A unified unit reporting protocol will allow healthcare providers, constantly and correctly, to interpret the results and avoid misdiagnosing iatrogenic hypermagnesemia, and it will facilitate reporting and exchange of results among the different laboratories.

## Introduction

Monitoring magnesium and magnesium sulfate blood levels are crucial to avoid iatrogenic hypermagnesemia [1]. Hypermagnesemia is a rare disease usually seen in renal failure settings or an iatrogenic supplementation, for instance, over administration of magnesium sulfate [2]. Magnesium sulfate treats many conditions, such as hypomagnesemia, prevents seizures in preeclampsia, and manages seizures in eclampsia patients [3]. Many units are used to report the magnesium level and the therapeutic range of magnesium sulfate, such as mEq/L, mg/dL, and mmol/L. Based on the unit, the therapeutic range of the magnesium sulfate can be 4.0-7.0 mEq/L, 4.8-8.4 mg/dL, or 2.0-3.5 mmol/L [3].

Additionally, monitoring the magnesium and the magnesium sulfate therapeutic level while administering the drug is essential; especially when factors leading to hypermagnesemia are present such as hypothyroidism, Addison's disease, familial hypocalciuric hypercalcemia, lithium therapy, laxative, and antiacid-containing magnesium [4]. Considering that hypermagnesemia can be lethal, as indicated by signs and symptoms such as prolonged PR interval and QRS duration, increase in QT interval, complete heart block, respiratory depression, hypocalcemia, and neuromuscular toxicity [5].

An underestimation or overestimation of the magnesium level may occur when healthcare providers are subjected to different reporting units used by different laboratories, which increases the possibility of interpretation error. Therefore, it is necessary to standardize these units across the College of American Pathologists (CAP) and Clinical Laboratory Improvement Amendments (CLIA) accredited laboratories.

## Case presentation

A 32-year-old female patient (G4P4004) at 31 weeks with a history of a multi-substance use disorder was transferred to the hospital after a presumed opiate overdose and acute hypoxic respiratory failure. She was found near a dumpster after taking 2.5mg of fentanyl. En route to the first hospital ER, she received midazolam and haloperidol for agitation. Then, she started escalating oxygen requirements from around 77% on room air to 95% on 10L on the nonbreathing bag, up to 50L on high flow nasal cannula. Her serum toxicology screen was positive for amphetamines, cocaine, opiates, benzodiazepines, fentanyl, and cannabis. She required maximum doses of dexmedetomidine with occasional midazolam of 4-6 mg for sedation. Her blood pressure and her liver function test increased. Her albumin was 3.4 g/dl, aspartate transaminase (AST) was 104 IU/l (normal range 10-42 IU/L), alanine transaminase (ALT) was 19 IU/l (normal range 6-45 IU/L), and anaplastic lymphoma kinase (ALK) phosphatase was 148 IU/l (normal range 34-104 IU/L); however, the renal function test was within the normal range. She displayed several spasmodic movements. Her troponin I was elevated at 648 ng/L (normal range 3-37 ng/L), and her B-type natriuretic peptide was 186.1 pg/mL (normal range 0.0-176.0 pg/mL), but her ECG was normal. Magnesium sulfate was initiated on a 6 mg bolus, followed by 2 mg/dl due to preeclampsia concerns. Then, she was transferred to an intensive care unit at another hospital, where she was intubated.

The hospital to which she was taken to reported magnesium and magnesium sulfate levels in mEq/L. The magnesium sulfate level reached 8.2 mg/dL. The travel nurse did not alert the physician at this hospital as magnesium sulfate was still within with therapeutic range of 4.8-8.4 mg/dL. However, considering that 4.0-7.0 is the therapeutic range of magnesium sulfate utilizing mEq/L as a reporting unit, the magnesium sulfate level was higher than the therapeutic level for the second hospital. Therefore, the physician discounted the magnesium sulfate.

The patient also was found to have normocytic normochromic anemia. Her hemoglobin was 10.9 g/dl, her hematocrit was 33.8 %, her mean corpuscular volume (MCV) was 92.5 fL, and her mean corpuscular hemoglobin (MCH) was 29.5 fL. Her platelets were within a normal range (169 10e9/L). Her temperature was elevated (96 F) with increased white blood cells (17.7 10e9/L). Chest radiograph showed diffuse bilateral infiltrates. However, a transthoracic echocardiogram showed a dilated inferior vena cava and mild pulmonary hypertension. Given the drop in hemoglobin from 10.9 g/dL to 9.8 g/dL in one day and the possible rapid development of pulmonary infiltrates, she was diagnosed with aspiration pneumonia and was started on vancomycin and meropenem. She also was found positive for treponema pallidum antibody, and she was treated empirically with intramuscular penicillin G 2.4 million units. The fetus was delivered via Cesarian section on the third day of admission in the setting of persistently low fetal heart rates. The patient was extubated on the fourth day of admission. Treatment of pneumonia and diuresis for intravenous Lasix was continued, and the patient was weaned to a 4L nasal cannula. The patient was deemed appropriate for transfer back to the ward and later discharged on the fifth day.

## Discussion

The reason for reporting this case is to highlight the differences in protocols for reporting magnesium and magnesium sulfate units and how misinterpreting them can place the patient in potentially disastrous clinical scenarios [6]. Reporting units can be expressed as mEq/L, mg/dL, or mmol/L. The normal ranges for these units are 1.3-2.1 mEq/L, 1.56-2.52 mg/dL, or 0.65-1.05 mmol/L. In the case of administration of magnesium sulfate, the therapeutic range would be 4-7 mEq/L, 4.8-8.4 mg/dL, or 2.0-3.5 mmol/L [7].

**Table 1:** Different magnesium normal ranges and magnesium sulfate therapeutic range reporting units

Unit	Magnesium normal ranges	Magnesium sulfate therapeutic ranges
mEq/L	1.3-2.1	4.0-7.0
mg/dL	1.56-2.52	4.8-8.4
mmol/L	0.65-1.05	2.0-3.5

In our case, the physician began magnesium sulfate as a part of the management plan for preeclampsia [8]. One of the healthcare team members, the travel nurse, thought the reporting unit in the second hospital was also mg/dL while it was actually mEq/L. As a result, the nurse did not report it as the magnesium sulfate was within therapeutic range as measured with mg/dL; subsequently, the physician was not alerted. However, as the hospital to which the patient was brought used the unit of mEq/L, the magnesium level appeared to have exceeded the normal range, which could have put the patient in a hazardous clinical scenario. As a result, the physician stopped the magnesium sulfate. Our clinical chemist then explained the difference between the reporting units to the healthcare team and alerted them. Such an incident highlights the importance of unifying the reporting units across the laboratories accredited by the CAP and the CLIP to eliminate confusion and misinterpretation among clinicians and healthcare providers.

## Conclusions

Magnesium sulfate is one of the drugs used for preeclampsia management. It prevents the patient from developing seizures. Monitoring the magnesium sulfate level is essential to avoid iatrogenic hypermagnesemia. Hypermagnesemia appears with different symptoms, such as hypotension, headache, and cardiovascular and neurological sequelae. In reporting the magnesium and magnesium sulfate level, laboratories use different units such as mEq/L, mg/dL, and mmol/dL. Although these units are accurate, utilizing many of them at the same laboratory or across different laboratories can lead to physicians and other healthcare providers misinterpreting the result. These errors can lead to misdiagnosing of iatrogenic hypermagnesemia. Therefore, we highly recommend standardizing units across laboratories. Unified reporting protocol will yield many advantages, such as facilitating the workload, transferring the care of the patients, and avoiding medical errors. Clinicians and healthcare staff will correctly interpret the results and avoid missing hypermagnesemia. Moreover, it will facilitate reporting and exchange of results among the different laboratories.
