# Correction: Iatrogenic Hypermagnesemia in a Patient With Preeclampsia Caused by Misinterpretation of the Magnesium Reporting Unit Following Magnesium Sulfate Administration

**DOI:** 10.7759/cureus.c89

**Published:** 2023-01-04

**Authors:** Mohamed S Omer, Syed Latif, Ricky Grisson

**Affiliations:** 1 Pathology and Laboratory Medicine, Rhode Island Hospital, Brown University, Providence, USA

This article has been corrected to fix several incorrectly written measurements as detailed below. Both Cureus and the authors regret that these errors were not identified and corrected prior to publication.

Abstract: "Her troponin I was high at 648 mg/L" - "mg/L" changed to "ng/L"

Discussion: "The normal ranges for these units are 1.3-2.1 mEq/L, 0.65-1.05 mg/dL, or 1.7-2.2 mmol/L." changed to "The normal ranges for these units are 1.3-2.1 mEq/L, 1.56-2.52 mg/dL, or 0.65-1.05 mmol/L.

Discussion, Table 1: Values in column labeled Magnesium normal ranges changed as follows: "1.7-2.2" changed to "1.3-2.1", "0.65-1.05" changed to "1.56-2.52", "1.3-2.1" changed to "0.65-1.05"

